# Supplementary material for: Are carbon emissions trading and green financial instruments synergistic? -Comprehensive quantitative research based on content analysis
Source: PLoS One. 2024 Mar 7;19(3):e0298601. doi: 10.1371/journal.pone.0298601 (PMC10919601; doi:10.1371/journal.pone.0298601)
Supplement: S1 Data — (ZIP) [file pone.0298601.s001.zip › Data/地级市Z_F工作报告.zip!/╣π╢1⁄2/╣π╓▌╩╨/└┤╫╘╣1⁄2╓┌║┼í╛┬φ┐╦╩2╛▌═°í┐_2009.docx]

各位代表：

　　我代表广州市人民政府，向大会作工作报告，请予审议，并请政协委员和其他列席人员提出意见。

　　一、2008年工作回顾

　　过去的一年，是很不寻常、很不平凡的一年，我市遭遇历史罕见的低温雨雪冰冻和强台风强降雨等严重自然灾害，受到国际金融危机的严重冲击。面对严峻形势，我们坚持以邓小平理论和“三个代表”重要思想为指导，认真贯彻落实科学发展观，紧紧围绕建设全省“首善之区”的战略目标，团结依靠全市人民，同心同德，奋力拼搏，克服困难，经济社会保持良好发展态势，人民生活持续改善，较好地完成了市十三届人大三次会议确定的目标任务。

　　（一）经济保持平稳较快增长。面对急剧变化的经济形势，我们科学决策，及时采取了18项扩大内需和一系列支持企业发展的政策措施，促进经济平稳较快增长。全市实现地区生产总值8216亿元，增长12.3%。人均生产总值81233元，增长10%。源于广州地区的财政总收入达2477亿元，增长17.1%；其中地方财政一般预算收入622亿元，增长18.7%。全社会固定资产投资2105亿元，增长12.9%。社会消费品零售总额达3140亿元，增长21%。对外贸易稳步增长，广州地区完成进出口总值820亿美元，增长11.5%。其中出口430亿美元，增长13.4%。全市实际使用外资38亿美元，增长10.6%。利用外资结构不断优化，大项目带动特点显著，累计批准1000万美元以上的大项目达197个。在广州投资的世界500强企业达到169家。民营经济实现增加值2829亿元，增长12.5%，占全市GDP的比重达34.4％。尤其是民营工业逆势而上，全市规模以上民营工业完成产值1543亿元，增长17.3％，高于全市工业增速4.2个百分点。消费市场持续畅旺，物价过快上涨势头得到遏制，城市居民消费价格总水平同比上涨5.9%。

　　（二）现代产业体系建设取得积极进展。确立了构建以服务经济为主体，现代服务业、高新技术产业和先进制造业融合互动、协调发展的现代产业体系，重点推进“区域性现代服务业中心、国际商务会展中心、亚洲物流中心、华南科技创新中心和国内先进制造业基地”建设。出台《关于加快发展现代服务业的决定》及《关于加快推进广州市产业转移和劳动力转移的实施意见》，进一步调整优化产业布局，完成了琶洲员村地区、城市新中轴线区域、白鹅潭经济圈和白云新城及周边地区等四个重点功能区规划，加快了中心城区产业“退二进三”和产业、劳动力“双转移”步伐，服务业发展水平明显提升。全市第三产业实现增加值4849亿元，增长13.6%，占GDP比重达59.02%，对全市经济增长的贡献率达64.6%。我市被国务院认定为“中国服务外包示范城市”。南沙保税港区获国务院批准设立。地方金融机构改革取得阶段性成效，历史遗留问题逐步化解，珠江新城金融商务区集聚效应进一步增强，全市金融业增加值达384亿元，增长6.2%。联邦快递亚太转运中心建成投入运营，白云国际机场旅客吞吐量达3341万人次，增长7.9%。广州港货物吞吐量达3.47亿吨，增长1%，居全国港口第4位。其中集装箱吞吐量达1100万标箱，增长18.9%，跻身全球集装箱港口10强。国际会展中心二期和三期投入使用，会展业发展环境进一步优化。全市批发零售业商品销售总额达13428亿元，增长22.1%，广州“千年商都”优势进一步凸显。旅游业总收入突破800亿元，来广州旅游观光客人持续增长。汽车、石化、造船、钢铁以及重大成套和技术装备等先进制造业集群发展，工业加快向高科技、高集聚、高附加值升级转型。全市完成工业总产值12639亿元，增长13.1%。其中，三大支柱产业完成产值4509亿元。轿车产量达87.94万辆，增长12.3%，跃居全国轿车产区第一；自主品牌汽车研制取得新进展。电子信息产品制造业产值突破1000亿元，增速达17.7%。出台了《关于大力推进自主创新加快高新技术产业发展的决定》及配套措施，大力支持软件动漫、电子信息、环保节能、生物医药等一批重大科技专项，加快推进高新技术产业园区建设，高新技术产业加速发展。全市规模以上工业高新技术产品产值3981亿元，增长18.8%，高于全市规模以上工业增长5.6个百分点。年末，三次产业的比重为2.04：38.94：59.02。产业结构的不断调整优化，增强了我市经济抗风险能力，在保增长中发挥了重要作用。

　　（三）城市建设和管理有新进步。深化城市总体发展战略规划，新一轮城市总体规划和土地利用总体规划编制工作全面展开。以交通为重点的重大基础设施建设抓紧推进，白云机场二期、南沙港区三期和出海航道疏浚三期、广州新客站站场及武广客运专线等项目进展顺利。34项道路、隧道、桥梁等工程以及7条地铁线路加快建设，快速公交系统试验段动工建设。完成了我市历史上最大一轮公交线网调整优化工作，方便了市民出行。从去年开始政府分三年每年出资4.63亿元补贴公交企业;出资18亿元,分三年补贴公交地铁票价。出台8项措施，规范了出租车行业管理。新增停车泊位超过6万个。建成了一批电力、供水、供气、环卫和人防工程设施。“青山绿地”和“蓝天碧水”二期工程建设取得新成效，全市林木绿化率达44.4%，森林覆盖率达38.2%，人均公共绿地面积13.01平方米，荣获“国家园林城市”和“国家森林城市”称号。为改善人民群众关心的水和空气环境质量，制定了《污水治理和河涌综合整治工作方案》，继续加大力度推进大规模的治理水环境行动，污水处理系统和西江引水、北部水系补水等具有重大意义的工程建设取得积极进展。制定了《2008-2010年空气污染综合整治实施方案》，实施七个方面50项污染防治措施，特别是强化工业企业和机动车排气污染防治，促进空气环境质量继续改善。全年空气质量优良天数比例达94.3%，比上年增加3个百分点。气象部门监测的灰霾天气天数由2007年的131天减少到110天，下降16%。认真落实节能减排目标责任制和考核制度，突出抓好百家重点耗能工业企业节能减排工作，关停13家燃油小火电、66家小水泥企业和140家挂牌督办违法排污企业。预计全市万元生产总值能耗下降4.35%，完成省下达的年度主要污染物二氧化硫和化学需氧量总量分别减排0.5万吨的任务，排放总量分别下降4.8%和3.7%以上。加强国土资源管理，开发补充耕地66.2平方公里，耕地实现占补平衡，整顿违法用地取得明显效果。进一步整顿和规范市场经济秩序，严厉打击制售假冒伪劣商品、传销、商业欺诈、无证照经营等违法行为，尤其是加大对乳制品市场的监管力度，高效稳妥处置“问题奶粉”事件，不仅保证了产品质量和本地食品、药品安全，而且保证了我市供应北京奥运会的食品安全。探索建立城市管理长效机制，启动星级卫生街道创建活动，市容环境卫生进一步改善。继续推进社区人居环境综合整治，加快了“城中村”和旧城区改造，完成13.9万平方米危破房改造，1800多户居民受益。社会治安综合治理进一步加强，“六好”平安和谐社区建设全面推进，共安装25.6万个监控摄像头，治安视频监控实现全市城区全覆盖，社会治安形势持续好转。去年刑事案件下降13.9%，其中“两抢”、“两盗”案件分别下降25.5%和17.3%，群众对社会治安的满意度持续上升。大力开展安全生产“隐患治理年”活动，进一步强化重点行业、重点领域安全监管和消防安全工作，安全生产形势保持稳定，全年事故起数、死亡人数分别下降16.7%和13.5%。

　　（四）农村、农业和农民工作保持良好发展势头。认真贯彻落实中央一号文件和党的十七届三中全会精神，进一步加大强农惠农工作力度，实现农业平稳增长，农民收入持续增加，农村社会保持稳定。全市实现农业总产值290亿元，增长3%；农业增加值168亿元，增长1.8%。农民人均纯收入9828元，增长14.1%。都市型现代农业建设稳步推进，水利设施、农田、鱼塘标准化改造和规模养殖场建设取得新进展。农业生态观光旅游发展势头良好。农业龙头企业和农民专业合作社不断发展壮大。落实促进农民增收的各项政策措施，农业补贴标准逐步提高，补贴范围逐步扩大。对5.6万名农村富余劳动力进行了职业技能培训，全年转移就业7.2万人。中心镇建设取得新进展，人口与产业集聚条件不断改善。安排6000万元推进镇村医疗卫生机构改造建设，完善农村卫生服务体系，每个镇拥有了1所以上政府兴办的卫生院。农村合作医疗制度逐步完善，参合率达99.4%。“万村千乡”市场工程建设基本完成，农村生活消费品、生产资料连锁销售网络和药品监管供应网络已覆盖全市行政村，农村消费环境进一步改善。稳步推进村庄规划编制工作，加大村容村貌整治力度。村务民主管理工作扎实推进，完成了村委会换届选举。

　　（五）重点领域改革取得新进展。为进一步转变政府职能，促进城市可持续发展，在交通、水务、地铁、燃气、环卫等城市建设领域组建7个专业投资集团，迈出了城市投融资体制改革的新步伐。推进城管体制改革，整合管理和执法职能，建立了城管局。制定实施城市建设维护工作市、区分工方案，进一步提高了城市建设和管理水平。水务管理体制改革顺利推进，基本实现了政府对水务的统一规划、建设和管理。文化体制改革取得新突破，为文化事业和产业发展创造了有利条件。中心镇行政执法体制改革试点稳步推进。着眼增强国有企业竞争力，加快股份制改革步伐，国有企业整体改制取得新突破，国有经济结构进一步优化。全市90%的国有大中型企业完成公司制改造，初步构建起新型国有资产监督管理体系，国有资产运行质量逐步提高。

　　（六）抗灾救灾和支援地震灾区工作成绩突出。去年初发生的低温雨雪冰冻灾害给春运工作造成了前所未有的困难，我们采取前所未有的措施，连续奋战九昼夜，夺取了抗灾保春运的决定性胜利。春运结束后，又认真总结经验教训，修改完善春运工作四级应急预案，努力提高政府应对复杂局面和处置突发事件的能力。五六月间，我市又接连遭遇五次强降雨强台风袭击。面对重大灾情，我们全力组织抗洪抢险和灾后复产工作，把损失降到了最低程度。四川汶川发生特大地震后，我市迅速响应中央和省的号召，组成7支专业救援队伍赶赴灾区展开紧急救援。全市人民奉献爱心，募捐善款16.21亿元，物资价值1.41亿元，创历史纪录。同时,接收救治灾区重伤员175名,按时保质完成了中央和省下达的建设6376套板房的任务。抗震救灾转入恢复重建阶段后，我市又派出前线工作组和专责小组，对口支援汶川县城威州镇重建家园、恢复生产、发展经济。首期总投资6亿元的11个援建项目已经启动。

　　（七）各项社会事业取得新成绩。亚运会筹备工作扎实推进，广州亚运城市行动计划进入全面实施阶段。开展了“还我蓝天迎亚运”和“迎亚运、讲文明、树新风、促和谐”等活动。新建改建场馆陆续动工，亚运城正加紧施工，场馆周边及主干道两边环境整治已经启动。加快推进义务教育规范化学校建设，首批25所示范性普通高中创建学校通过省的终期督导验收。教师待遇进一步改善。全面实施城乡免费义务教育，积极做好扶困助学工作。设立专项资金，推动民办教育和职业教育取得新发展。我市成为省教育强市，教育整体实力迈上新台阶。组织发动全市力量推进全国文明城市创建活动，开展12项专项行动，城市文明程度和市民素质进一步提高。社会主义荣辱观教育不断深化，我市被中央文明委命名为首批“全国未成年人思想建设工作先进城市”。大力发展志愿服务事业，志愿工作蔚然成风。文化事业不断进步，市、区、街（镇）、社区（乡村）四级文化设施网络进一步健全。广州国际艺术博览会、羊城国际粤剧节、广州民俗文化节等一批重要文化活动项目成为我市文化品牌。新河浦历史文化保护项目获得“世界大都市奖”第二名，成为我国内地首个获此殊荣的项目。完成了《粤剧大辞典》的编纂出版。纪念改革开放30周年系列文艺活动、“四进社区”群众文艺大汇演等活动和免费开放一批博物馆、纪念馆，受到群众欢迎。知识产权管理和保护工作取得成效，我市成为首批国家“知识产权工作示范城市”。新闻出版和广播电视事业健康发展，文化市场进一步规范。体育强市建设扎实推进，群众体育活动日益普及。我市运动员在北京奥运会勇夺首金并创历史最好成绩，成功举办世乒赛等52项国际和全国体育大赛。多个国际体育组织在广州设立机构，体育产业加快发展。人口计生工作“升类创优”活动全面开展，优质服务覆盖面进一步扩大，奖励优惠政策得到进一步落实，人口自然增长率为4.7％。。社区服务取得良好效果，“平安通”呼援服务系统覆盖全市老城区。认真落实老年人优待政策，我市连续第四次获全省老龄工作目标责任考核第一名。国防后备力量工作不断加强，“防空防灾一体化”民防体制建设扎实开展。我市连续第七次被命名为“全省双拥模范城”。妇女儿童、残疾人事业取得新成绩。民族团结进步事业深入开展，宗教界参与公益慈善事业作出了新贡献，在维护社会稳定中发挥了积极作用。区域经济协作和对口帮扶工作取得积极进展，泛珠三角省会城市合作进一步深化。打击走私和海防建设不断加强。“信息广州”建设取得新进展，我市被批准为国家首批移动电子商务试点示范城市，市政府网站绩效在全国同类城市评估中名列前茅。气象基础设施建设稳步推进，天气预报准确率不断提高。积极开展国际交流与合作，我市获中国人民对外友好协会授予的“友城交流合作奖”；行政首长首次以世界城市和地方政府联合组织联合主席身份主持国际会议，提高了广州的国际声誉和知名度。统计、审计、侨务、对台、档案、保密、修志、参事、文史、科普等工作进一步加强。

　　（八）民生福利进一步改善。在继续落实66条惠民措施基础上，市委、市政府又出台了17条补充意见，着力解决群众关注的热点问题，进一步扩大了群众受惠覆盖面。制定实施被征地农民、城镇老年居民、农村居民养老保险和城镇居民、农转居人员医疗保险等政策措施，实现了养老和医疗保险制度覆盖全体城乡居民。调升了全市56万多名退休人员基本养老金，企业退休人员月人均养老金水平达到1563元，增长20.51%。提高了工亡补助金和伤残津贴等工伤保险待遇。外来工得到生育保险保障。最低工资标准由780元调整为860元，城市低保标准从330元提高到365元，农村低保标准平均水平从202元提高到220元。社会救助、社会福利、优抚安置水平进一步提升。统筹做好城乡新增劳动力就业、失业人员再就业和外来劳动力转移就业工作，采取多项措施促进广州生源高校毕业生就业，登记在册的“零就业家庭”一人以上就业率达100%，全市新增城镇就业人数25.7万人，城镇登记失业率为2.32%，就业形势保持基本稳定。加快建设社区卫生服务机构，公共医疗卫生服务体系逐步完善，社区药品统一配送和医疗机构药品网上限价竞价阳光采购工作稳步推进，群众看病贵问题有所缓解。继续加强保障性住房建设。通过入户调查确定了到2012年基本解决77177户低收入家庭住房困难问题的目标，去年共推出2145套经济适用房，以廉租房实物配租或租金补贴形式，为2102户新增“双特困”家庭和14649户城市低收入困难家庭提供了住房保障。人民生活水平进一步提高，全市职工年人均工资达45251元，增长12.6%，城市居民年人均可支配收入25317元，增长12.7%。为更好地倾听民声、了解民意、排解民忧，我们创新信访工作机制，开展市、区、街（镇）三级公开大接访活动，有效解决了一大批群众反映的突出问题和实际困难。积极开展法律援助，法律服务和普法工作得到加强，人民调解和排查调处工作机制不断完善，成功预防和化解了大批矛盾纠纷，维护了社会和谐稳定。

　　（九）政府自身建设进一步加强。坚持定期向人大通报重要工作，自觉接受市人大及其常委会的监督，主动接受市政协的民主监督。积极听取各民主党派、工商联和人民团体的意见。人大代表建议和政协提案777项全部办复，办理情况得到人大代表和政协委员的肯定。坚持实施《行政许可法》等法律法规和规章制度，政府法制工作取得新进展，决策和运行机制进一步健全，行政行为进一步规范。稳步推进财政投资评审工作，节省了财政资金。建成市政务服务中心，31个部门和机构入驻，为市民和企业提供行政许可及配套服务事项783项，行政管理效率和服务水平进一步提高。制定了行政机关及其公务员公共服务行为投诉处理办法和首问首办责任制，促进了机关作风建设和公务员队伍建设。扎实推进绩效考评、行政监察、审计监督，坚持不懈地开展反腐败斗争，政风行风和勤政廉政建设取得良好成效。

　　各位代表，过去的一年，我们经受了异常严峻的考验，取得了来之不易的成绩，这是省委、省政府和市委正确领导的结果，是全市人民团结奋斗和各方面支持帮助的结果。在此，我代表广州市人民政府，向全市人民、向驻穗人民解放军、武警部队官兵表示崇高的敬意！向关心和支持广州建设的港澳台同胞、海外侨胞、国际友人表示衷心的感谢！

　　在总结成绩的同时，也应清醒地看到，我市经济社会发展仍存在一些突出问题。发展方式尚未根本转变，自主创新能力不够强，城乡发展还不平衡，面临的人口、资源、环境压力加大。城市建设、管理和社会发展存在一些薄弱环节，创建全国文明城市工作与市民群众普遍满意的要求仍有差距。去年下半年以来，国际金融危机对我市实体经济的影响逐步显现，外部需求萎缩，经济增长放缓，企业效益下滑，财政增收困难，就业压力加大。我们要正视存在的问题和困难，进一步做好各项工作。

　　二、2009年工作安排

　　今年是广州经济社会发展面临更大困难和挑战的一年，也是蕴含更大希望和机遇的一年。我们既要保持清醒头脑，增强忧患意识，又要坚定信心，迎难而上，积极应对国际金融危机。要深入贯彻落实科学发展观，贯彻中央经济工作会议和省委十届四次全会精神，深刻认识《珠江三角洲地区改革发展规划纲要》（下称《纲要》）的重大意义并狠抓贯彻落实，坚持以《纲要》为行动纲领，以省委、省政府“三促进一保持”的要求为工作主线，全力推动广州经济社会又好又快发展，加快建设全省“首善之区”，强化国家中心城市、综合性门户城市和区域文化教育中心地位，增强高端要素集聚、科技创新、文化引领和综合服务功能，以广佛同城化为突破口，促进珠三角一体化发展，在全省实践科学发展观中当好排头兵。

　　今年经济社会发展主要预期目标是：地区生产总值增长10%；人均生产总值增长8.5 %；全社会固定资产投资增长12.5%；社会消费品零售总额增长13%；外贸出口总值力争有所增长；万元生产总值能耗下降4%，二氧化硫和化学需氧量排放总量分别下降5%和3.9%；城市居民人均可支配收入增长9%；农村居民人均纯收入增长9%；居民消费价格总水平涨幅控制在4%以内；城镇登记失业率控制在3.6%以内；人口自然增长率控制在6.3％。以内。重点抓好以下九项工作：

　　（一）立足扩大内需，努力实现经济平稳较快增长。

　　保增长是今年的首要任务，扩大内需是保增长的根本途径。我们要按照《纲要》要求，抓紧落实中央和省关于扩大内需的各项部署，抓住筹办亚运会的重大机遇，打好“亚运牌”，加快形成主要依靠内需拉动经济增长的格局，同时努力保持出口和利用外资规模，促进经济平稳较快发展。

　　充分发挥投资对经济的拉动作用。抓紧建设一批对经济社会长远发展关系重大的项目。今明两年安排重大建设项目投资约3500亿元，带动全社会固定资产投资约5000亿元。其中，今年安排82个重点项目，计划投资806.5亿元，重点投向四个方面：一是推进亚运场馆、亚运城和相关配套设施等亚运工程建设；二是机场、港口、地铁、高快速道路等重大城市基础设施建设；三是与建设现代产业体系相关的物流、商贸、会展、汽车、电子、石化、重型装备、生物医药、新材料和新能源等重大产业项目；四是民生工程建设，包括生态环境保护、农村道路、水利设施、商业网点、现代农业示范区以及森林公园保护性开发等基础设施建设，以及教育、医疗、文化、住房保障等。要调动多种投资主体的积极性，以政府投资带动社会投资，提高投资效益，达到既促进经济发展,又保障和改善民生的目的。

　　积极扩大消费需求。正确引导市民消费预期，完善和落实消费政策，优化消费环境，增强消费品市场活力。保持房地产市场平稳健康发展，稳定房地产开发投资规模。落实国家住房消费优惠政策，支持居民合理住房消费。培育汽车、假日、服务等消费热点，扩展电子商务、网络教育、文化娱乐及体育健身等新兴消费领域，推动消费模式的升级换代。进一步发展旅游业，创新旅游产品，策划推出广州岭南古城历史文化之旅，打造广府文化生态旅游黄金线路，加快建设旅游强市。积极开拓农村消费市场，完善“万村千乡”市场网络，推进“家电下乡”工作，提高农民消费能力。

　　力争进出口和利用外资有所增长。创新对外贸易发展方式，实施多元化市场战略，引导企业调整出口目标、开拓新兴市场。优化调整出口结构，提高产品附加值。加快推进广州服务外包示范城市建设，积极承接国际服务业外包，促进贸易转型升级。发挥外向型企业资助资金作用，加快出口退税进度，充分发挥保税物流特殊监管区的政策功能优势，推动设立白云空港综合保税区，为外贸企业发展创造有利条件。创新对外投资与合作方式，加强与世界主要经济体的经贸关系，积极主动参与国际分工与合作。支持企业拓展国际市场，扩大境外加工贸易和合作开发资源。深化穗港澳台合作，落实CEPA系列协议。加大招商引资力度，创新招商引资方式，大力引进重大生产力项目，尤其是现代服务业项目，吸引外资重点投向先进制造业和高新技术产业。

　　优化经济发展的服务环境。密切关注国内外经济环境和宏观政策的变化，及时掌握重点区域、重点行业、重点企业发展情况，增强经济运行调节的科学性、预见性和有效性。各级政府、各部门要落实和细化国家和省、市关于促进经济增长、扶持企业发展的优惠政策，主动为企业提供服务，协调解决生产经营和重大项目建设中存在的突出问题，与企业共渡难关。千方百计做好各项生产保障工作，促进重要生产要素供需平衡。加快清除企业发展的障碍，法律法规未禁止的行业和领域均向各类经济主体开放。依法清理涉及企业的行政事业性收费，改革堤围防护费征收，阶段性大幅度降低失业保险、城镇职工基本医疗保险及工伤保险的缴费比例，切实减轻企业负担。

　　（二）加快转变发展方式和结构调整，增强经济抗风险能力和国际竞争力。

　　抓住国际经济结构深刻调整的机遇，以转变发展方式和调整结构为主攻方向，按照《纲要》要求,推进重大产业基地和重点项目建设，加快产业优化升级，提高现代服务业占服务业的比重，提高高新技术产品产值占工业总产值的比重，提高先进制造业产值占工业总产值的比重，提高民营经济占全市经济总量的比重，为抵御经济风险奠定坚实基础。

　　着力构建现代产业体系。加快建设国家级信息化和工业化融合试验区，推动现代服务业、高新技术产业和先进制造业融合互动、协调发展。重点推进琶洲员村地区、城市新中轴线区域、白鹅潭经济圈和白云新城及周边地区等四个服务业发展重点功能区建设，推进广州（黄埔）临港商务区规划建设，促进现代服务业集聚发展。构建多样化、多层次的金融综合服务体系，完善金融产业政策，加大珠江新城金融商务区建设力度，推进区域金融中心建设。发展和利用资本市场，积极推进企业上市融资。建设国际商务会展中心，打造国际一流的会展品牌。积极推进批发市场升级改造，加快建设广州国际商品展贸城，规划利用流花交易会旧馆，优化流花商圈，促进现代展贸业发展。建设十三行商圈，提高传统商贸业水平。着眼建设世界一流物流中心，加快白云机场和南沙港等枢纽型现代物流园区建设，抓紧完善与现代物流业相配套的基础设施。充分发挥联邦快递亚太转运中心、广州保税物流园区和南沙保税港区在珠三角区域经济一体化中的辐射带动作用，强化大宗原材料交易中心功能，加快形成大宗商品的“广州价格”。大力发展总部经济，培育发展创意产业。做优做强先进制造业。全力建设汽车、石化、造船、电子信息四大国家级产业基地。规划建设具有国际先进水平的广州大型装备产业园区，推进省市共建先进制造业基地建设。加快发展自主品牌乘用车，提高参与国际汽车市场竞争的能力。为促进传统产业转型升级，加快黄埔石化厂改扩建的选址工作，推进中科合资广东南沙炼化一体化项目建设，优化我市石化产业布局，减少污染物的排放。大力发展船舶工业和海洋工程设备、核电设备制造业及其他重大成套数控装备，加快推进龙穴造船和大岗低速船用柴油机项目建设，打造具有国际竞争力的高端制造业基地。运用先进适用技术改造传统产业。强化自主创新，壮大高新技术产业。围绕建设华南科技创新中心和成果转化基地，进一步完善自主创新体制机制和政策环境，强化企业技术创新主体作用，鼓励和支持企业建设技术研发中心，实施引进、消化、吸收再创新示范工程，推动产学研联合。加快国家级数字家庭应用示范产业园区建设，集中力量抓好电子信息产业、现代信息服务业、生物产业、装备制造业、新材料、数字家庭和数字电视六大科技专项，力争在关键技术领域取得新突破。实施“自主创新一区一成果行动计划”，从今年起，市科技经费每年安排6000万元，对12个区（县级市）各支持1个高水平科技成果转化，加快高新技术产业园区、广州科学城北区、国际生物岛和国家级电子信息、软件动漫、数控装备及生物四大产业基地建设，培育大型高新技术企业集团。建设一批公共创新平台，继续办好中国留学人员广州科技交流会，培育和引进高层次、国际化科技创新人才。

　　促进民营经济和中小企业发展壮大。要在政策支持、产业引导和社会服务上加大力度，把民营经济培育成为广州最具活力的经济体。加强广州民营科技园建设，鼓励引导民营企业优化结构，提升创新能力，培育一批竞争力强的龙头骨干企业。针对当前的经济困难，加大对中小企业的扶持力度，搭建银企合作平台，完善担保体系，创新融资产品，切实解决中小企业融资难等问题。实施“中小企业成长工程”，设立创业投资引导基金。进一步加强中小企业管理机构建设，强化中小企业综合性、社会化服务。

　　切实抓好节能减排和节约集约用地工作。各级政府要带头树立节约资源意识，严格落实节能减排问责制和“一票否决制”。按照新的产业结构调整指导目录，严把项目准入关。加快淘汰能耗高、污染大的企业，推行绿色制造和清洁生产，着力发展循环经济。培育环保产业，开发新能源，推广应用节能节水技术和产品。鼓励企业加快节能节水降耗技术改造。要高度重视土地资源的开发管理，科学编制土地出让和储备计划，盘活存量土地。积极推动地下空间开发，提高土地科学利用率。完善土地利用综合评价标准，严把节约集约用地关口，严肃查处违法用地行为。

　　积极推进“双转移”和中心城区产业“退二进三”。加强产业转移规划引导，实行产业转移和企业升级并举。推动一批发展前景良好的“退二”企业入驻承接产业基地，引导一批不适宜在我市发展的企业向市域外转移，促进工业向园区集中。加快实施“腾笼换鸟”战略，对腾出的工业厂房，在规划允许的情况下，调整功能，进行内部改造，合理利用，鼓励发展工业设计、建筑设计、产品展示及商贸、餐饮、会议、住宿、休闲娱乐等服务业。加快产业转移园区建设，完善“双转移”和产业“退二进三”优惠政策，加强企业职工技能培训和转岗培训，调动企业转移的积极性和主动性。

　　（三）以建设宜居城市为目标，全面提高城市建设和管理水平。

　　树立城市以环境论输赢的观念，坚持一手抓经济发展，一手抓环境建设，努力提升城市发展的承载力，进一步改善环境质量，以崭新的城市形象迎接2010年亚运会。

　　加强城市规划和城市基础设施建设。全面完成新一轮城市总体规划和土地利用总体规划编制工作，加快编制主体功能区规划，促进三个规划有机衔接。抓紧编制控制性详细规划，力争实现全市总体规划范围内控制性详细规划全覆盖。以《纲要》提出的建成珠江三角洲地区一小时城市圈的核心为目标，加快推进白云国际机场扩建工程、武广铁路及新客运站相关工程、南沙港区三期及广州港出海航道三期等一批重大枢纽性交通基础设施建设。全力推进在建7条地铁线路建设，确保五号线建成通车，力争启动六号线二期、七号线、九号线建设。进一步完善高快速道路网络，重点推进广深沿江、广河、广明、东新、增从、大广等高速公路建设。全面推进快速公交系统建设，力争年底投入运营；继续加快公交枢纽站和公交地铁综合换乘站的建设，新增停车泊位5万个。完成黄埔大道支线、广州大道改造等一批市政道路和桥梁工程，改善交通节点，优化人行过街设施，方便群众出行。

　　综合整治水环境和空气环境。把改善水和空气环境作为重大的民生工程，作为建设“首善之区”的重大任务，举全市之力，多管齐下，综合整治，到亚运会召开前实现全市水环境和空气环境明显好转。系统推进污水处理系统建设、河涌综合整治、调水补水、中心城区雨污分流、水浸街改造和依法从严控制污水排放六大任务。继续抓好工业企业、机动车、饮食服务业、工地扬尘等七个方面50项污染防治工作，进一步改善空气质量。在加快脱硫工程建设的同时，推进火力发电企业脱硝工程建设。全面实施机动车环保标志管理。进一步研究灰霾天气形成和消散机理，在省的支持协调下，与周边城市一齐行动，努力减少灰霾天气。

　　加快实施花园城市建设行动纲要。巩固扩大创建国家园林城市和国家森林城市成果，推进森林围城和生态保护计划。重点建设火炉山、帽峰山、王子山、天鹿湖等8个森林公园和自然保护区，加大万亩果园、南沙湿地等6个湿地公园保护建设力度，推进采石、采泥场复绿和矿山地质生态环境恢复工作。进一步完善城市总体绿化布局，推进亚运城、亚运场馆周边地区和主要道路、城市主要出入口和重要地标周边的绿化景观升级改造；完善城乡公共绿地系统，加强城市社区、河涌沿岸、工业园区和新农村绿化建设，促进城乡绿化一体化，重塑广州“花城”形象。

　　大力推进旧城区改造和社区人居环境综合整治。在加强历史街区整体保护的同时，加快旧城区和“城中村”改造。重点推进恩宁路、东濠涌、南华西等旧城区改造和猎德村、杨箕村、花地村等“城中村”改造项目。完成120个社区人居环境综合整治任务。在全市社区全面推进“五个一”工程，即每个街道建设和完善一个服务中心、一个卫生服务机构、一个小公园、一个群众娱乐场所、一个治安视频监控中心，构建完善的社区服务体系，努力改善市民的生活条件。

　　进一步提高城市管理水平。着力解决城市管理的突出问题，促进城市管理科学化、规范化和精细化。实施商品交易市场信用分类监管，认真吸取“瘦肉精”中毒事件教训，完善产品、食品、药品安全和整规打假长效监管机制，建立严格的检验制度，加强安全风险动态监测和执法，确保产品质量和食品、药品安全，保障人民身体健康。加强城市综合执法，完善市容环境卫生长效管理机制，依法严厉整治和清除“六乱”。加强道路交通组织管理，改善交通状况。严格落实安全生产责任制，严防重特大安全生产事故发生。

　　（四）大力统筹城乡发展，加快形成城乡经济社会发展一体化新格局。

　　认真贯彻落实中央和省关于全面推进农村改革发展的要求，大力推进城乡一体化发展，建立健全以工促农、以城带乡的长效机制，促进公共服务在城乡之间均衡配置、生产要素在城乡之间自由流动，使广大农民平等参与现代化进程，共享改革发展成果。

　　大力建设都市型现代农业。着眼于构建现代农业产业体系，推进农业设施化、集约化、科技化和标准化建设，完善城乡水利防灾减灾工程体系。积极发展生态农业、观光农业和出口创汇农业，推动乡村旅游、森林生态旅游和“农家乐”、“林家乐”加速发展，促进农民增收。加强农业科技创新及推广体系建设，扶持农业龙头企业发展，延伸现代农业链条，发展农产品加工业，提高农民从事二、三产业的比例，增加农民工资性收入和非农产业经营性收入。落实各项强农惠农政策措施，增加农民补贴性收入。建立新型农业社会化服务体系，推进大型农副产品批发市场建设，促进农村流通现代化。加快发展农民专业合作社，提高农民的组织化程度和农业抗击风险、参与市场竞争的能力。

　　全面推进农村改革。制定实施相关配套政策，创新、完善农村基本经营制度和土地制度，促进农村土地承包经营权和农村集体建设用地流转，实现农民土地合法权益财产化，增加农民财产性收入。积极稳妥开展林权制度改革，让林农得到实惠。深化征地制度改革，完善补偿机制。探索宅基地置换机制，统筹规划农村住宅建设，妥善解决农民建房问题。健全农业投入保障模式，建立政府引导、多方投入的村级公益事业建设新机制。探索建立现代农村金融制度，加快农业政策性保险试点工作。改革中心镇财政体制和行政执法体制，支持中心镇发展。健全农村民主管理制度，切实维护农民合法权益。

　　积极发展农村各项社会事业。切实提高财政对农村公共事业的投入，着力完善农村教育、卫生、文化、体育等公共服务体系。加快城乡交通设施和水、电、气设施一体化建设步伐，进一步改善农民生产生活条件。加强农村生态环境保护，完成全市1138个行政村的规划编制，开展试点村、重点村村容村貌整治工作，促进社会主义新农村建设。加强农村社会管理，广泛开展创建文明镇、文明村、文明户活动，开展农村社区建设实验工作。引导城市志愿者服务农村，推进农村扶贫开发，支持北部山区经济社会协调发展。

　　（五）推进重点领域的各项改革，着力构建有利于科学发展的体制机制。

　　增创体制机制新优势，是破解发展难题，实现新发展的关键。要毫不动摇地坚持改革方向，用好《纲要》给予的先行先试政策，抓住时机，深化重点领域和关键环节改革，增强经济社会发展的生机与活力。

　　稳步推进行政管理体制改革。重点做好新一轮政府机构改革工作。完善行政运行机制，提高政府依法行政水平和管理服务效能。加快政府职能转变，深化行业协会、商会改革，理顺政府、社会、市场以及政府部门之间的关系。创新政府管理方式，启动第四轮行政审批制度改革，大幅度精简审批项目。在机关事业单位实行机构编制实名制管理。全面推进事业单位分类改革。推进行政事业性收费改革，规范收费项目。深化城市管理体制改革，推动管理权限进一步向区（县级市）下放。

　　深化经济领域改革。以完善公司治理机制为重点，深化国有企业改革。继续推进和深化国有企业公司制股份制改革，鼓励整体改制、整体上市。完善董事会制度、监事会制度，探索建立职业经理人制度。加快国有资本结构调整和国有企业重组，促进国有资本向优势产业、优势企业和优势产品集中。按照“责、权、利”相统一和“管资产与管人、管事相结合”的原则，理顺国有企业领导人管理体制，健全企业负责人激励约束机制，完善国有资产管理体制。深化财政体制改革，完善市对区的财政体制。继续推进城市投融资体制改革，培育壮大投融资主体，充分发挥投融资集团的作用。深化广州市商业银行等地方金融机构改革，积极推进期货市场发展，加快国家级区域性产权交易中心建设。加快广州市农村信用合作社改制为农村商业银行步伐。推进营商标准与做事规则国际化的制度创新，促进广州经济与国际市场接轨。

　　积极探索社会管理体制改革。学习借鉴发达国家和地区的先进经验，推进社会管理改革创新先行先试，逐步构建党委领导、政府负责、社会协调、公众参与、社会化市场化运作的新型社会管理体制，提高社会公共服务质量和水平。探索公立医院管办分开、规范运行的体制，改革以药补医机制。抓紧实施文化体制改革，促进文化事业和文化产业的新发展。加快社会工作人才队伍建设，推进社会工作发展。鼓励社会组织参与社会管理，提供公共服务。完善社区自治体制，增强基层自治能力。

　　（六）以强烈的责任感和紧迫感，全力推进亚运会筹备工作。

　　以省委汪洋书记提出的“迎接亚运会，创造新生活”为主题，按照办一个创新、廉洁、勤俭的运动会的要求，精心策划，周密部署，全面实施亚运城市行动计划，全力做好筹备工作，把亚运会办成展示广东和广州改革开放形象、高水平、有特色的亚洲体育盛会。

　　要立足城市长远发展，统筹推进亚运城、运动场馆及配套设施建设，综合整治场馆周边和主干道两侧环境，建设和完善城市光亮工程，美化城市环境。广泛开展“迎亚运、讲文明、树新风、促和谐”活动，持续创建全国文明城市，提升城市形象，提高市民素质，实现“2010年一大变”。抓紧制定和完善亚运会交通通信、安全保卫、环境保护、食品安全、医疗卫生、气象服务等各项保障方案。实施城市志愿者行动，做好赛时各类志愿者的招募、培训。加大亚运城市宣传推广和市场开发力度，创新全民动员形式，提高全民参与效果。同步推进广州亚残运会的筹备工作。

　　（七）大力加强社会建设，促进各项社会事业全面进步。

　　强化社会建设意识，着眼于提高城市文明水平，满足人民群众日益增长的物质文化需要，大力发展社会事业，促进人的全面发展。

　　努力提升文化软实力。加强社会主义核心价值体系建设，加强理想信念和经济形势任务教育。大力弘扬新时期“敢为人先、奋发向上、团结友爱、自强不息”的广州人精神。发挥哲学社会科学界“思想库”作用，为经济社会发展提供智力支持。制定和实施《2009－2011年创建全国文明城市规划纲要》，全面动员部署新一轮创建全国文明城市工作，把创建工作不断引向深入。加快建设广州新图书馆、歌剧院、新电视塔等一批大型城市标志性文化设施。推进南越王宫博物馆、辛亥革命纪念馆等重点文化设施和惠及市民的公共文化设施建设，广泛开展“文化进社区”和“文化下乡”公益活动。精心筹划、加紧筹备第九届中国艺术节，以办好中国音乐金钟奖、中国国际漫画节、中国（广州）国际纪录片大会、声响亚洲艺术节以及广州国际艺术博览会、羊城书展等文化活动为依托，打造优秀文化品牌。加强对历史文化资源的挖掘、保护和开发利用工作，积极推进广州海上丝绸之路文化遗址申报世界遗产。着力发展文化创意产业，打造具有广州特色的文化产业体系，进一步净化文化市场环境。加强知识产权管理与保护，形成全社会尊重和保护智力成果的良好氛围。

　　继续实施教育强市战略。健全义务教育经费保障机制和监督机制，落实教育惠民措施，推动公共教育资源向困难地区、困难学生和困难群众倾斜，完善扶困助学制度，认真解决进城务工农民子女接受义务教育问题，促进教育公平。加快义务教育规范化学校建设，充分发挥示范性普通高中的示范辐射作用，进一步提升市属高校办学水平。积极发展职业教育和民办教育。加强教师队伍建设，切实改善教师待遇。

　　促进其他各项社会事业新发展。加快推进社区体育设施建设和公共体育设施向社会开放工作，广泛开展全民健身活动，提高人民群众健康水平。全力办好2009年苏迪曼杯世界羽毛球混合团体赛等国际和全国体育大赛，积极开展亚运体育项目推广活动，促进体育产业加快发展。完善人口计生利益导向政策体系，加强计划生育公共服务网络建设和流动人口计划生育服务管理，稳定低生育水平。认真实施妇女儿童发展规划，切实维护妇女儿童合法权益。积极构建养老服务体系，加快发展养老服务事业。贯彻党的民族宗教工作基本方针政策，促进民族团结，发挥宗教在促进社会和谐中的积极作用。加强国防动员和后备力量建设。继续抓好“双拥”优抚安置工作，巩固军政军民团结。加强海防建设,坚决打击走私行为。加强防空防灾设施建设，促进人民防空向防空防灾相结合的民防体制转变，推进人民防空事业新发展。加快气象监测网络和监测预警中心建设，提高天气预报和灾害性天气预警能力。认真做好第二次经济普查工作。加强外事、侨务、港澳台工作，不断扩大对外交流与合作，认真筹办2009年在我市举行的世界城市和地方政府联合组织世界理事会以及国际友好城市大会。重视加强科普工作，继续做好档案、保密、修志、参事、文史等工作。

　　（八）着力保障和改善民生福利，切实维护社会和谐稳定。

　　经济发展越困难，越是要高度关注民生民情，及时解决群众的切身利益问题。要切实把66条惠民措施和17条补充意见落到实处，多为群众办实事、办好事，努力做到保民生，促和谐。今年，年度预算专项资金要削减10%，同时严格控制党政机关一般性行政经费支出，公务购车和用车经费、会议经费、公务接待费用、党政机关出国（境）费用以及办公经费预算实行“五个零增长”，拿出更多资金增加改善民生的投入。

　　务必做好就业工作。要把就业放在保民生的首位，实施更加积极的就业政策，营造全社会关注、支持就业的社会环境。全力促进就业增长，确保就业形势稳定。积极推进创建充分就业社区工作，加强失业动态监测，重点做好困难群体的就业援助工作，力争实现持证失业人员就业率70%以上，登记在册“零就业家庭”1人以上就业率100%，无连续失业1年以上人员。重视劳动力结构的调整和劳动力素质的提高，大力发展职业技能培训教育，探索“以创业培训带动就业”的新路子。制定鼓励和支持广州生源高校毕业生灵活就业和自主创业的措施，为高校毕业生就业创造更宽松的环境。加强对企业劳动关系的指导和服务，建立健全工资分配利益均衡机制，构建和谐劳资关系。加大劳动保障监察执法力度，妥善调解劳资纠纷，切实维护劳动者合法权益。

　　着力构建共享型社会保险、社会救助和社会福利体系。推进城镇老年居民、被征地农民和农村社会养老保险扩面工作。积极稳妥地开展事业单位养老保险制度改革。实施城镇职工基本医疗保险普通门诊统筹制度。出台外来从业人员医疗保险办法，实施养老、失业、工伤保险市级统筹，提高失业保险保障水平。继续提高企业退休人员基本养老金水平。切实做好困难群体、老年群体、优抚群体等保障工作。实施农村低保救助、低收入困难家庭救助和困难群众重大疾病医疗救助“三大工程”，调整提高城乡低保标准和低收入困难家庭认定标准，逐步扩大社会福利的惠及范围。

　　加快完善医疗卫生服务体系。调整医疗卫生资源的空间布局，加快卫生资源相对薄弱区域的配套建设，逐步实现医疗卫生资源的合理配置。鼓励和规范社会力量办医，提高医疗资源利用效率。认真落实关于发展城市社区卫生服务的实施意见及配套政策，加快完善城市社区卫生服务网络，健全农村三级医疗卫生服务体系。加强中医医疗服务体系和基层中医院建设。继续做好重大疾病防控、卫生应急工作，加强医疗卫生服务监管和药品价格管理，努力减轻群众就医负担。

　　进一步提高住房保障水平。健全住房保障制度，完善保障性住房财政投入政策和优先供地机制，加快廉租房、经济适用房建设，确保在2012年底前使全市77177户在册的城市低收入住房困难家庭的住房“应保尽保”。合理调整建设规模，完善分配政策，确保保障性住房的公平性。

　　扎实做好社会稳定工作。深入开展“六好”平安和谐社区建设，改进出租屋和流动人员、外籍人员的服务管理，做好刑释解教人员和社区矫正对象的帮扶。切实加强社会治安综合治理，保持严打高压态势，严厉打击多发侵财犯罪、严重暴力犯罪和毒品犯罪。创新完善动态打防机制，促进社会治安形势进一步好转。健全社会矛盾调处机制，加大社会矛盾纠纷排查化解力度，推动人民调解、行政调解、司法调解相衔接，提高突发性群体性事件的处置能力。

　　继续做好支援汶川县威州镇灾后恢复重建工作。坚持在受援地党委、政府的统一领导下，抓紧建设和修复与群众生活紧密相关的居民住房、公共服务设施和基础设施，帮助灾区恢复生产、发展经济、重建家园。不折不扣地完成省下达的任务，努力把威州镇建设成为灾区恢复重建的样板。

　　（九）加强政府自身建设，进一步提高依法行政能力和公共服务水平。

　　面对新的形势和任务，各级政府必须加强自身建设，努力提高贯彻落实科学发展观的能力，为全面完成各项工作任务提供坚强有力的保障。

　　认真落实社会主义民主政治制度。自觉接受同级人大及其常委会的法律监督和工作监督，重视政协的民主监督，定期向人大报告工作和向政协通报情况，认真执行人大及其常委会的决议、决定和审议通过的议案有关处理意见，及时办理人大代表建议和政协委员提案。完善民主决策、依法决策、科学决策机制，坚持专家咨询、社会公示和听证制度。充分发挥民主党派、工商联、无党派人士、政府参事的参政议政作用；认真听取工会、共青团、妇联等人民团体的意见，充分发挥其联系群众的桥梁纽带作用。不断巩固和扩大基层民主，保证人民群众依法行使民主权利。

　　强化依法行政和公共服务。各级政府要强化法制观念，自觉运用法律手段调节、管理经济和社会事务，做好行政立法、行政执法监督、行政复议工作，严格按照法定权限和程序行使职权、履行职责。更加注重公共服务和社会管理，维护社会公正和社会秩序，促进基本公共服务均等化。进一步完善市政务服务中心建设，改进公共服务方式，优化服务流程，推行联合审批、一站式服务、限时办结和承诺服务。建立完善区（县级市）政务服务中心和镇（街）便民服务中心。积极实施政务公开，健全政府信息发布制度，增强政府工作透明度，营造公开、公正、规范、便捷的行政环境。加快推行电子政务，进一步提高行政效率。

　　加强政风行风和廉政勤政建设。各级领导干部要自觉学习实践科学发展观，始终保持积极进取的精神状态，切实转变工作作风，敢于直面矛盾，攻坚克难，真抓实干。完善领导干部联系和服务群众的工作机制。推行政府绩效管理和行政问责制，完善部门之间协调配合机制，形成干事创业的强大合力。坚持求真务实，精简会议和文件，强化督促检查，狠抓工作落实。继续开展民主评议政风行风活动。加强公务员队伍教育、培训和管理，促进首问首办责任制落实，提高公共服务质量。加强廉政勤政建设，深入推进反腐倡廉工作，治理商业贿赂，强化经济责任审计，逐步推进审计结果公开，坚决惩治腐败。倡导勤俭节约，反对奢侈浪费。

　　各位代表，新的一年，我们面对新的形势、新的任务。我们坚信，任何困难都阻挡不了我们前进的步伐，沧海横流更显出广州这座英雄城市的本色。让我们更加紧密地团结在以胡锦涛同志为总书记的党中央周围，在市委的正确领导下，高举中国特色社会主义伟大旗帜，以邓小平理论和“三个代表”重要思想为指导，深入贯彻落实科学发展观，万众一心，开拓奋进，为加快建设全省“首善之区”，以优异成绩迎接新中国成立六十周年，开创广州更加美好的未来而努力奋斗！
